# Template-Choice Bias in Deep Sequencing of Antibody Phage Display Libraries

**DOI:** 10.3390/biom16101379

**Published:** 2026-09-22

**Authors:** Hyunji Yang, Hwa Kyoung Lee, Taehoon Ryu, Duck-Kyun Yoo, Junho Chung

**Affiliations:** 1 Department of Biochemistry and Molecular Biology, College of Medicine, Seoul National University, Seoul 03080, Republic of Korea; jk05025@snu.ac.kr; 2 Cancer Research Institute, College of Medicine, Seoul National University, Seoul 03080, Republic of Korea; 3 Department of Biomedical Science, College of Medicine, Seoul National University, Seoul 03080, Republic of Korea; intia6@gmail.com; 4 ATG Lifetech Inc., A-909, Lionsvalley, 168, Gasandigital 1-ro, Geumcheon-gu, Seoul 08507, Republic of Korea; thryu@atglifetech.com

**Keywords:** phage display, bio-panning, phagemid sequencing, antibody discovery, antibody engineering

## Abstract

Background/Objectives: Deep sequencing of antibody phage-display selections commonly uses intracellular double-stranded phagemid DNA (dsDNA), although selected libraries are propagated as virions containing single-stranded phagemid DNA (ssDNA). Whether these two templates yield equivalent quantitative profiles remains uncertain. Methods: We sequenced matched intracellular dsDNA and virion ssDNA from a single-chain variable fragment library at the input and after each of four rounds of bio-panning. Results: A total of 1052 ssDNA-derived and 1093 dsDNA-derived unique heavy-chain complementarity-determining region 3 (HCDR3) clonotypes were analyzed, including 1003 shared clonotypes. The dsDNA and ssDNA profiles were closely concordant before strong enrichment, with Spearman correlations of 0.972–0.978 and Morisita–Horn overlap of 0.994–0.998 through round 2. Once a small number of clonotypes began to dominate the selection, however, the two profiles separated sharply: abundance-weighted overlap fell to 0.501 at round 3 and 0.379 at round 4, whereas rank order was preserved and the rank correlation remained high throughout (ρ ≥ 0.90). This divergence was concentrated among enriching trajectories, in which dominant clonotypes reached substantially higher relative abundance in virion ssDNA than in intracellular dsDNA. Antigen-reactive clonotypes were also preferentially shifted toward the ssDNA-biased fraction, with a median log_10_(ssDNA/dsDNA) ratio of 0.44 compared with −0.17 for non-reactive clonotypes at round 3 (*p* = 6.3 × 10^−8^) and round 4 (0.41 vs. −0.12, *p* = 1.1 × 10^−6^). Conclusions: dsDNA and ssDNA tell nearly the same story early in bio-panning but can provide markedly different accounts once selection becomes concentrated. In this selection campaign, template choice materially affected interpretation of antibody phage-display sequencing, suggesting that virion ssDNA or paired-template analysis may be preferable to dsDNA alone when late-round abundance is used for candidate prioritization. Because these results come from a single campaign against one antigen, they are descriptive of this dataset and require validation across antigens, scaffolds and display systems.

## 1. Introduction

Phage display technology was developed by G. Smith in 1985 as a powerful method for presenting polypeptides or proteins on the surface of filamentous bacteriophage [1]. The inherent biology of phage, wherein genotype and phenotype are physically linked, makes this technology directly applicable to drug discovery. Specifically, the gene encoding the displayed molecule is copied and packaged within the same virion as a single-stranded DNA (ssDNA), and the displayed protein is expressed as a fusion to a phage coat protein [2]. This genotype–phenotype linkage ensures that all progeny phage particles produced from a single *Escherichia coli* (*E. coli*) clone carry an identical, clone-specific fusion protein. This phage display technology has conferred the ability to construct antibody libraries encoding 10^6^–10^11^ different ligands within a population of >10^12^ displayed phage particles. Through bio-panning, desired fusion phages can be enriched more than 1000-fold over background phage, using repeated cycles of incubation, washing, amplification, and re-selection of bound phage [3].

Conventionally, bio-panning has been referred to as the procedure for selecting specific binders based on their affinity toward a target. In fact, however, two distinct implications are conflated in this procedure: first, bio-panning provides affinity-based enrichment [4,5]; and second, it enriches for “good expressors”, clones that tend to both propagate preferentially in the *E. coli* host and package efficiently into bacteriophage particles [6,7,8]. Therefore, under both target-binding selection and amplification/fitness-driven selection pressures, two confounding biases arise during bio-panning. First, a gradually increasing bacterial amplification bias, caused by the metabolic burden of the inserted antibody library, gives rise to parasitic sequences that persist through later rounds of bio-panning [7,9]. This bias favors fast-growing sequences; as a result, “slow-growing” binders are consistently lost from the selection, while “parasitic” clones tend to be enriched regardless of their target-binding ability [10]. Second, differences in phage packaging efficiency that depend on antibody sequence or structure can cause compositional bias [11,12]. Examples include Sec pathway censorship and the formation of intermolecular disulfide bonds between unpaired cysteines in the antibody sequence and the intrinsic cysteine of pIII, both of which can interfere with phage assembly or infectivity [13,14,15]. In this regard, bio-panning is a selective enrichment procedure driven not only by affinity selection but also by phenotypic fitness selection.

Conventional approaches, such as Sanger sequencing of a limited number of individual clones or enzyme-linked immunosorbent assay (ELISA)-based screening, sample only a small fraction of the library and are therefore poorly suited to detecting the subtle, round-by-round shifts in clonal composition.

Previously, 454 pyrosequencing of amplified phage particles was also used to analyze phage enrichment at a much larger scale, more quickly, and more accurately [16]. More recently, high-throughput sequencing (HTS) has enabled large-scale parallel analysis, allowing researchers to screen for positive binders not only at the end of bio-panning but also to monitor library composition and diversity at each round throughout the procedure [17,18,19,20]. One aspect that has been overlooked, however, is that in the majority of cases, phagemid double-stranded DNA (dsDNA) is sequenced rather than the bacteriophage’s actual single-stranded DNA, an approach that relies on the implicit assumption that the dsDNA repertoire closely reflects the ssDNA repertoire of the phage-displayed library. These two templates, however, are not simply two preparations of the same material. The replicative-form phagemid reflects the composition of the bacterial population in which the library was amplified, whereas the packaged ssDNA reflects the composition of the particle pool that is physically carried into the next round of selection; a discrepancy between them is therefore a property of the phage life cycle itself, rather than of the DNA extraction procedure. In cases where bacterial amplification bias and packaging-efficiency-related compositional biases exist, the composition of enriched bacterial dsDNA and bacteriophage ssDNA may diverge as bio-panning proceeds. However, no study has performed a parallel comparison of dsDNA and ssDNA sequences across all bio-panning rounds.

In this article, we analyzed heavy-chain complementarity-determining region 3 (HCDR3) sequences of dsDNA (bacterial plasmid) and ssDNA (bacteriophage) from four rounds of bio-panning (Round 1–4) of a single-chain variable fragment (scFv) phage-display library to conduct a side-by-side comparison. Among the three HCDR3 clonotype trajectories identified—enriching, intermediate, and depleted clusters—ssDNA HCDR3 clonotype frequency was overrepresented relative to dsDNA within enriching clusters, and this bias became more pronounced as bio-panning progressed.

## 2. Materials and Methods

### 2.1. Library, Bio-Panning, and Template DNA Preparation

The starting material was a chicken (*White leghorn*) immune scFv phage-display library against recombinant mouse c-Met-Fc chimera protein (R&D Systems, Minneapolis, MN, USA; Cat. No. 527-ME) as described for chicken combinatorial scFv libraries [21] using standard phage-display methodology [22]. All animal procedures were approved by the Ethics Committee of BioPOA Ltd. (Hwaseong, Republic of Korea; ethical approval code: BP-2019-C03-1). Selection used four rounds of bio-panning on antigen-conjugated magnetic beads [22,23], giving fractions R0 (the pre-panning library input) through R4. Output/input panning titers were measured at each round as described previously [22].

Each round was sequenced from two DNA template preparations made in parallel from the same panning batch. dsDNA, the double-stranded replicative-form phagemid, was prepared from the infected *Escherichia coli* (*E. coli*) pellet with the QIAprep Spin Miniprep Kit (QIAGEN, Hilden, Germany; Cat. No. 27104); ssDNA, the single-stranded phagemid packaged inside M13 phage particles, was isolated from the culture supernatant with the QIAprep Spin M13 Kit (QIAGEN, Hilden, Germany; Cat. No. 27704), each according to the manufacturer’s instructions.

### 2.2. Sequencing and Read Processing

Both template repertoires were sequenced on a HiSeq 2500 instrument (Illumina, San Diego, CA, USA) using a HCDR3-focused short amplicon. Because chickens carry essentially a single functional germline heavy-chain variable (VH) and heavy-chain joining (JH) gene segment, the heavy-chain framework regions are near-invariant and repertoire diversity is concentrated in the HCDR3. A custom reference of approximately 200 chicken VH sequences (assembled from NCBI records and the authors’ own clones) was used to identify the most conserved framework region 3 (FR3) and framework region 4 (FR4) segments, against which the amplification primers were placed; the resulting approximately 130-bp amplicon spans the 3′ end of FR3, the complete HCDR3, and the 5′ start of FR4. The primers were those described previously for the same library [21]: HFR3 5′-GGCTGCAGCTGAACAACCTCAGGGCTG-3′ and HFR4 5′-GGAGGAGACGATGACTTCGGTCCCGTGG-3′. For each template and round, 100 ng of phage ssDNA or phagemid dsDNA was amplified with 2.5 pmol of each primer in a 50 µL reaction using KAPA HiFi HotStart DNA polymerase (KAPA Biosystems, Roche, Basel, Switzerland) with the following thermal cycling program: 95 °C for 3 min; 17 cycles of 98 °C for 30 s, 65 °C for 30 s, and 72 °C for 1 min 10 s; and a final extension at 72 °C for 5 min. Amplicons were purified with SPRI beads (AMPure XP; Beckman Coulter, Brea, CA, USA), and the size distribution of each library was verified on a TapeStation instrument (Agilent Technologies, Santa Clara, CA, USA) with a D1000 ScreenTape assay (Agilent Technologies, Santa Clara, CA, USA) before sequencing.

Paired-end reads were merged with PEAR v0.9.6 using default parameters [24] and filtered to remove reads below the minimum Phred quality threshold, reads lacking the library-construction primer, out-of-frame reads, and reads without an identifiable HCDR3; the HCDR3 was delimited by its conserved FR3 and FR4 framework boundaries, and any HCDR3 clonotype supported by fewer than two reads was discarded. For the analysis set used in this study, an HCDR3 clonotype was defined as a unique HCDR3 amino-acid sequence within a given library that (i) passed a base-quality threshold of Phred Q ≥ 40 across the HCDR3 (basecall error rate ≤ 0.01%) and (ii) reached a depth-normalized abundance of at least 100 parts per million (PPM) in at least one round. Depth normalization was applied per library per round: for clonotype i at round r, PPM = (Q40-filtered reads of i at r)/(total Q40-filtered reads at r) × 10^6^, so that PPM ≥ 100 corresponds to ≥ 0.01% of reads. Q ≥ 40 was applied because the amplicon carries essentially all of its diversity in the HCDR3, so a single miscalled base creates a spurious clonotype; PPM ≥ 100 was applied because, below that level, a clonotype is supported by a handful of reads and its round-to-round trajectory is dominated by sampling noise. Re-running the whole pipeline at PPM cut-offs of 200, 500 and 1000 left the reported bias unchanged (enriching-cluster slope 0.52, 0.53 and 0.45 at R3 against 1.01, 0.99 and 1.02 at R2). The Q ≥ 40 quality threshold was assessed in the same way: re-running the analysis at Q ≥ 30 and Q ≥ 20 left the reported bias unchanged (enriching-cluster slope at R3 0.573 and 0.564 against 0.574 at Q ≥ 40, the lower threshold discarding 36% of the reads). After filtering, the ssDNA library contained 1052 clonotypes and the dsDNA library contained 1093 clonotypes, representing 1142 unique HCDR3 amino-acid sequences, of which 1003 were detected in both libraries.

### 2.3. Combined Clonotype Sets

Three combined representations of the two libraries were used, each chosen to fit the analysis. Because diversity is a property of unique clonotypes, the two libraries were merged into a single repertoire for the diversity metrics (the union: 1142 unique HCDR3 sequences, with the reads of shared sequences summed; Table S1). For enrichment clustering, the per-library tables were instead concatenated into 2145 clonotype-by-library rows, keeping each library’s copy of a clonotype separate so that its ssDNA and dsDNA versions could be clustered and labeled independently. Finally, because a paired ssDNA-versus-dsDNA comparison is defined only when both values exist, every such comparison used the clonotypes detected in both libraries (the per-round shared set; n = 1001 at R0–R1 and 1003 at R2–R4), which underlies the bias correlations and the antigen-reactivity analysis.

### 2.4. Enrichment Clustering and Correlation Analysis

For the pooled, ssDNA-only, and dsDNA-only sets, each clonotype’s five-round PPM trajectory (R0–R4) was z-scored row-wise across rounds and clustered by Ward hierarchical clustering [25] (scipy.cluster.hierarchy.linkage with method = ‘ward’, partitioned with fcluster using criterion = ‘maxclust’) at k = 3. Clusters were labeled by their mean change in z-score from R0 to R4 [mean (z at R4–z at R0)]: C1 (enriching cluster) the highest, C3 (depleted) the lowest, and C2 (intermediate, peaking at R2) in between. On the pooled table, the cluster sizes were C1 = 491, C2 = 264, and C3 = 1390 (sum 2145); on the ssDNA-only set, in the same order, 160/75/817; and on the dsDNA-only set, 262/191/640. k = 3 was set a priori to represent the three trajectory classes expected in a selection experiment (enriching, transient and depleted) rather than chosen by optimizing an internal validity index. The enriching cluster is identical at k = 2 and k = 3 (the same 491 rows); only k = 2 and k = 3 are stable under bootstrap resampling (mean Jaccard 0.82 and 0.77 over 200 resamples, against 0.60–0.65 for k = 4–6), and the template bias reported below is unchanged for k = 2–5 (Figure S1C–E).

Template bias was quantified using the pooled cluster labels applied to the shared clonotypes. For each round and each cluster, the Spearman rank correlation (ρ) and the ordinary-least-squares slope of log_10_(dsDNA PPM) on log_10_(ssDNA PPM) were computed for clonotypes detected (PPM > 0) in both libraries; a slope < 1 indicates that ssDNA overrepresents its most abundant clonotypes relative to dsDNA. Because both axes carry sampling error, the slopes were also computed with standardized major axis (SMA) and Deming (orthogonal) regressions; these attenuate the effect less but give the same pattern, with slopes of 0.71 (SMA) and 0.65 (Deming) at R3, and 0.69 (SMA) and 0.63 (Deming) at R4, for the enriching cluster, against ≈1.0 for the other clusters in every round. The fits drawn in the figures and the slopes quoted in the Results, however, are the ordinary-least-squares estimates. Restricting a cluster to clonotypes present in both libraries retains only those whose ssDNA and dsDNA copies received the same pooled label (788 cluster-concordant of the 1003 shared at R2–R4: C1 = 146, C2 = 62, C3 = 580; the 215 cluster-discordant clonotypes are excluded from the colored per-cluster panels). The same ρ and slope computed over all 1003 shared clonotypes give the “all” curve, so the colored per-cluster (n = 788) and the “all” (n = 1003) curves differ in denominator.

### 2.5. Clone Retrieval and Phage ELISA

Individual clones corresponding to HCDR3 sequences identified by deep sequencing were physically retrieved from the last round of panning output by high-throughput single-clone retrieval (TrueRepertoire, TR; ATG Lifetech, Seoul, Republic of Korea) [26]. Single colonies were picked into 0.5 mL Super Broth (SB) medium (30 g/L tryptone, 20 g/L yeast extract, 10 g/L 3-(N-morpholino)propanesulfonic acid [MOPS]) supplemented with 100 µg/mL carbenicillin (Gold Biotechnology, St. Louis, MO, USA; Cat. No. C-103-50) in Axygen^®^ 96-well 2.2-mL polypropylene deep-well plates (Corning, Corning, NY, USA; Cat. No. P-2ML-SQ-C) and grown at 37 °C, 250 rpm for 3–4 h. Each well was superinfected with 100 µL VCSM13 helper phage (Agilent Technologies, Santa Clara, CA, USA; Cat. No. 200251, approximately 10^10^ pfu/mL) for 15 min at 37 °C; after a further 1.5 h, 100 µL SB supplemented with kanamycin and carbenicillin was added and the plates were incubated overnight at 37 °C, 250 rpm for phage production. Cells were pelleted at 3000× *g* for 15 min and 200 µL of each phage supernatant was mixed with an equal volume of 6% (*w*/*v*) bovine serum albumin (BSA) (Thermo Scientific, Rockford, IL, USA; Cat. No. 23209) in phosphate-buffered saline (PBS). High-binding 96-well plates (Corning, Corning, NY, USA; Costar 3590) were coated overnight at 4 °C with 100 ng/well recombinant mouse c-Met-Fc chimera protein (R&D Systems, Minneapolis, MN, USA; Cat. No. 527-ME) in coating buffer (0.1 M sodium bicarbonate buffer, pH 8.3). Wells that received 3% BSA/PBS only, with no antigen coated, served as the background control for every clone, and phage supernatant from a stored enriched panning round served as a positive control for detection. Plates were washed three times with 0.05% (*v*/*v*) Tween-20 in PBS (PBST), blocked with 3% BSA/PBS using 100 µL per well, incubated for 1 h at 37 °C, and washed again. Phage supernatant mixed with an equal volume of 6% BSA in PBS was applied to the wells at 100 µL/well for 1 h at 37 °C. After the plates were washed three times with PBST, horseradish peroxidase (HRP)-conjugated anti-M13 pVIII antibody (Sino Biological, Wayne, PA, USA; 11973-MM05) diluted 1:5000 in 3% BSA/PBS (100 µL per well) was added. After incubation for 1 h at 37 °C, the plates were washed three times. Then, 3,3′,5,5′-tetramethylbenzidine (TMB) substrate (Thermo Fisher Scientific, Waltham, MA, USA; 34029) was added at 100 µL per well. After incubation for 30 min at room temperature in the dark, the absorbance was measured using a Multiskan™ GO microplate spectrophotometer (Thermo Fisher Scientific, Vantaa, Finland).

A clone was scored antigen-reactive when its absorbance on the antigen-coated well exceeded the mean + 3 standard deviations (SD) of background control wells on the same plate as described previously [27]. In total, 692 clones distributed over eight 96-well plates were assayed, corresponding to 568 unique HCDR3 amino-acid sequences after collapsing wells that retrieved the same clone in duplicate.

### 2.6. Diversity, Overlap, and Antigen-Reactivity Mapping

Per-library, per-round clonal diversity was summarized by the Shannon entropy H [28], computed using the natural logarithm of the PPM-weighted clonotype frequencies; as a concentration metric, the cumulative read fraction of the top 1% of clonotypes was computed per library per round (reported as “Top-1%” in Figure 1A). Abundance-weighted overlap between the ssDNA and dsDNA repertoires was quantified per round with the Morisita–Horn index [29]. Directional cluster discordance among the 1003 shared clonotypes was assessed by cross-tabulating each clonotype’s ssDNA-copy and dsDNA-copy pooled labels into a 3 × 3 confusion matrix (rows = ssDNA-copy cluster, columns = dsDNA-copy cluster; Figure S3D). The read-weighted HCDR3 amino-acid length distribution was computed per library per round, with each clonotype contributing in proportion to its within-library read fraction (Figure 2C).

To assess whether the template bias affects antigen-reactive clonotypes, the phage-ELISA reactivity calls (determined as above) were intersected with the deep-sequencing analysis sets. The 692 assayed clones collapsed to 568 unique HCDR3 sequences; 538 were represented by a single well and 30 by two or more (up to 58 wells), of which 14 gave discordant well-level calls. A single per-HCDR3 call was therefore assigned by majority vote across its wells. The threshold was set at a mean call ≥ 0.7 at the outset, an arbitrary choice fixed before any analysis, so we varied it: only 14 of the 568 sequences have a mean call strictly between 0 and 1, and moving the cut-off from 0.6 to 0.9 therefore changes the number of antigen-reactive sequences only from 81 to 78, with the antigen-reactivity result at R3 and R4 significant throughout (*p* ≤ 0.002). The values reported here are those with a mean call ≥ 0.7: 90 antigen-reactive and 478 non-reactive sequences. Of these, 146 were present in both the ssDNA and dsDNA analysis sets (35 ELISA-positive, 111 ELISA-negative) and defined the universe for the reactivity panels (Figure 3A,B). Figure 3A overlays these on the per-round ssDNA-versus-dsDNA clonal-frequency scatter (all 1003 shared clonotypes in grey; ELISA-positive as filled purple, n = 35; ELISA-negative as open white, n = 111; red y = x reference line). Figure 3B compares the distribution of log_10_(ssDNA PPM / dsDNA PPM) at R3 between the ELISA-positive and ELISA-negative groups with a two-sided Mann–Whitney U test [30]. Because only HCDR3-matched clonotypes are available and there is no dsDNA-biased control group, this measures whether antigen-reactive clonotypes are enriched within the ssDNA-biased fraction, not whether the ssDNA/dsDNA bias predicts reactivity.

### 2.7. HCDR3 Sequence-Feature Analysis

To ask whether antigen reactivity is predictable from the HCDR3 sequence alone, the per-HCDR3 phage-ELISA calls determined above were used as labels for all 568 unique HCDR3 amino-acid sequences (90 antigen-reactive, 478 non-reactive). Four descriptive properties were computed per sequence: length; cysteine count, binned as 0, 1, or ≥2; mean hydropathy (grand average of hydropathicity, GRAVY) over residues on the Kyte-Doolittle scale [31] and net charge at pH 7, taken as the number of Lys and Arg residues minus the number of Asp and Glu residues. Antigen-reactive and non-reactive groups were compared for each property with a two-sided Mann–Whitney U test [30] (Figure S5A–D).

Two sequence representations were then tested. For the physicochemical representation, each residue was replaced by its five Atchley factors [32] and the factors were averaged over the sequence, giving a five-dimensional profile per clonotype. For the protein-language-model representation, sequences were embedded with ESM-2 (esm2_t12_35M_UR50D) [33] and the layer-12 residue representations were mean-pooled over the sequence, excluding the start and end tokens. Both representations were standardized feature-wise. These two analyses were restricted to the 562 sequences composed only of the 20 standard amino acids (88 antigen-reactive), excluding six sequences that contained an ambiguous or stop codon.

Separability was quantified with an L2-regularized logistic-regression probe (Atchley profiles, default regularization; ESM-2 embeddings, C = 0.1; maximum 2000 iterations), scored as the mean area under the receiver-operating-characteristic curve over repeated stratified five-fold cross-validation with 10 repeats and a fixed random seed. For visualization only, each representation was projected to two dimensions with Uniform Manifold Approximation and Projection (UMAP) [34] (n_neighbors = 20, min_dist = 0.3, fixed random seed) (Figure S5E,F); the projection itself was not used for any quantitative claim.

### 2.8. Statistics and Software

Clonal-frequency comparisons used Spearman rank correlations and ordinary-least-squares log-log slopes; group differences in log_10_(ssDNA/dsDNA) were tested with the two-sided Mann–Whitney U test [30]. All analyses were performed in Python 3.12 with NumPy 1.26, pandas 2.2, SciPy 1.13, Matplotlib 3.10, openpyxl 3.1, scikit-learn 1.8, umap-learn 0.5.11, fair-esm 2.0, and PyTorch 2.10. Because this study analyzed a single bio-panning campaign against a single antigen using HCDR3 sequence only, with no biological replicate, all statistics are descriptive of this dataset and are not used to claim generality.

## 3. Results

### 3.1. Generation of Scfv Phage Display Library and HTS Analysis of Extracted Phagemid

An immune scFv phage-display library with a complexity of 4.96 × 10^9^ transformants was constructed in the pComb3XSS phagemid vector from chickens (*White leghorn*) injected three times with recombinant mouse c-Met-Fc chimera protein [21,23,27]. At each round, two DNA templates were prepared in parallel from the same panning batch: the replicative-form phagemid recovered from the infected *E. coli* pellet (dsDNA) and the phagemid packaged inside M13 phage particles in the culture supernatant (ssDNA). Both were sequenced on an Illumina HiSeq 2500 instrument using an HCDR3-focused amplicon of approximately 130 bp.

### 3.2. ssDNA and dsDNA Templates Yield Concordant Bio-Panning Trajectories

We next compared the ssDNA and dsDNA template preparations across the four panning rounds to quantify any clonal bias between the two libraries. The two libraries were sequenced deeply, with 8.9 million ssDNA and 6.5 million dsDNA quality-filtered HCDR3 reads in the analysis set across the five rounds; the PPM ≥ 100 analysis set accounted for 27.8–59.6% of the quality-filtered ssDNA reads and 28.6–41.0% of the dsDNA reads (Figure S1A). All diversity, rank-correlation and abundance comparisons therefore use the per-library, per-round depth-normalized PPM values defined in the Methods rather than raw read counts. At this depth, the 1.4-fold difference between the two libraries does not affect the comparisons made here: rarefying the deeper library to the shallower one at every round, over 200 read-level subsamples drawn without replacement, left every reported statistic unchanged (at R3, enriching-cluster slope 0.574 and Morisita–Horn overlap 0.501), and at R3 and R4, the rounds at which the templates diverge, the dsDNA library is in fact the more deeply sequenced of the two. After filtering, the analysis set comprised 1052 ssDNA and 1093 dsDNA HCDR3 clonotypes, each defined as the set of reads sharing an identical HCDR3 amino-acid sequence (Figure S1A). Both template preparations captured the expected course of selection, with clonal diversity collapsing only at the late, enriched rounds. The output/input panning-titer ratio fell from 2.6 × 10^−4^ at Round 1 (R1) to 2.7 × 10^−7^ at Round 2 (R2), an approximately 960-fold drop, and then recovered to 4.5 × 10^−6^ at Round 3 (R3) and 4.6 × 10^−5^ at Round 4 (R4) under progressively more stringent washing (Figure 1A). Clonal diversity, measured as Shannon *H*, remained high and nearly identical between the two templates through R2 (ssDNA 6.12, 6.12, 6.04; dsDNA 6.17, 6.15, 6.03 at R0, R1, R2) and then collapsed at R3 (ssDNA 3.65; dsDNA 4.88), with the dsDNA repertoire re-diversifying slightly at R4 (ssDNA 3.96; dsDNA 5.35) (Figure 1A). Consistently, the read fraction held by the top 1% of clonotypes (the approximately 11 most abundant per library) rose from roughly 19–24% in the early rounds to 68.3% (R3) and 62.2% (R4) for ssDNA versus 44.7% (R3) and 37.5% (R4) for dsDNA (Figure 1A). The two templates thus agreed on the overall selection dynamics; the only divergence between them emerged in the later, more strongly enriched rounds (R3 and R4).

**Figure 1:**
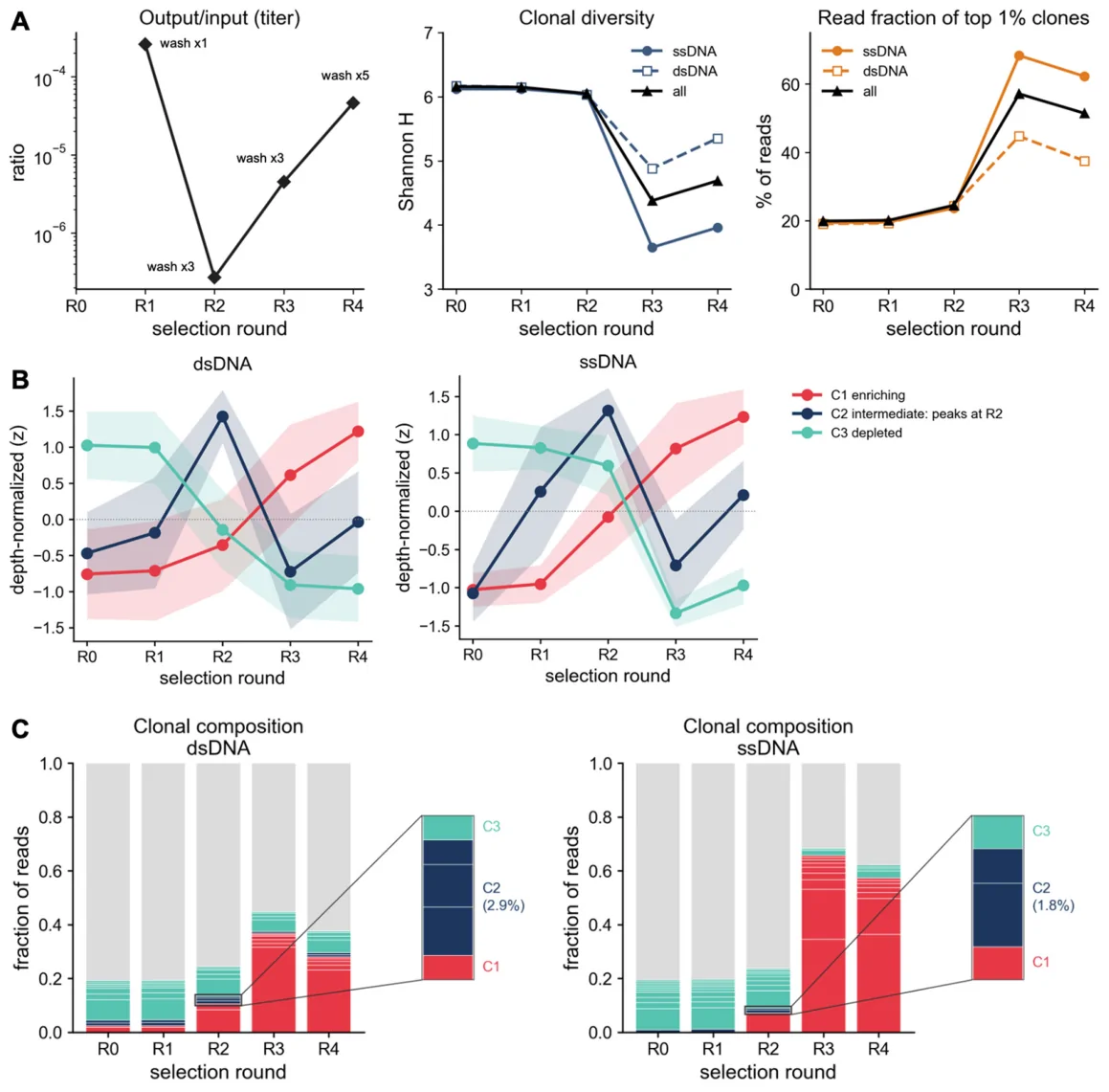
High-throughput sequencing (HTS) of dsDNA and ssDNA repertoires. (**A**) Per-round output/input panning titer ratio (left); clonal diversity (Shannon H); and read fraction of the most abundant (Top-1%) clones (right), all based on HCDR3 clonotypes. (**B**) Enrichment trajectories of the three clonal clusters resolved in each repertoire; dsDNA (left) and ssDNA (right) (Ward, k = 3, on z-scored per-round abundance; mean ± 1 SD plotted); C1 enriching, C2 intermediate: peaks at R2, C3 depleted. Cluster sizes (C1/C2/C3) are 262/191/640 for dsDNA (n = 1093) and 160/75/817 for ssDNA (n = 1052). (**C**) Clonal composition per round (stacked, fraction of reads); dsDNA (left) and ssDNA (right). The top 1% HCDR3 clonotypes in each repertoire, colored by cluster as in (**B**), all others grey.

Hierarchical Ward clustering (k = 3) of the z-scored five-round trajectories resolved the same three clonal trajectories in each library separately: an enriching cluster (C1), an intermediate cluster that peaks at R2 and then declines (C2), and a depleted cluster (C3), with cluster sizes (C1/C2/C3) of 262/191/640 for dsDNA (n = 1093) and 160/75/817 for ssDNA (n = 1052) (Figure 1B). Clustering the two libraries together, as 2145 clonotype-by-library rows, recovered the same three-trajectory structure (C1, n = 491; C2, n = 264; C3, n = 1390) and provided the common cluster labels used for the paired comparisons below (Figure 1B). The per-round clonal composition showed the same turnover at the read level, now resolved separately by template: each library’s own top 1% most abundant clonotypes were progressively taken over by C1 clonotypes, more completely in ssDNA (65.6% of all reads by R3) than in dsDNA (36.7%) (Figure 1C), paralleling the diversity collapse in Figure 1A. Per-library sequencing statistics (Figure S1A) were consistent with this picture (Figure 1A–C).

### 3.3. ssDNA Overrepresents the Most Enriched Clones at the Late Bio-Panning Rounds

To separate two conceptually different questions, whether the two templates agree on the relative ranking of clonotypes, and whether they agree on their absolute proportions, we used three complementary measures throughout this section: Spearman rank correlation (ρ, agreement on which clonotypes are more or less abundant, independent of scale), log-log regression slope (whether abundance scales proportionally between templates, with 1 meaning perfect proportionality), and the Morisita–Horn index (an abundance-weighted measure of how similar the two frequency profiles are overall, described further below). At the whole-library level, all three measures tracked closely between templates through R2; at the enriched rounds the rank correlation remained high while the slope and the abundance overlap diverged.

For the whole-library shared set, the ssDNA-versus-dsDNA rank correlation was very high through R2 (Spearman ρ 0.978, 0.978, 0.972 at R0, R1, R2), and the log-log slope, modestly below unity at R0–R1, reached unity by R2 (0.887, 0.897, 1.001). At the enriched rounds the rank correlation remained high (ρ 0.898 at R3, 0.943 at R4), whereas the slope fell (0.767 at R3, 0.791 at R4) (Supplementary Figure S2). Because the slope was already somewhat below 1 in the early rounds, it alone cannot establish that the templates are equivalent through R2—that claim instead rests on the consistently high rank correlation and on the abundance overlap (below). Against that baseline, the drop in slope specifically at R3–R4 indicates that ssDNA overrepresented its most abundant clonotypes relative to dsDNA.

Stratifying the shared clonotypes by pooled cluster localized this divergence almost entirely to cluster C1 (Figure 2A,B). C1 maintained very high rank correlation through R2 (ρ 0.952, 0.943, 0.936 at R0, R1, R2) with log-log slopes that were modestly below or at unity but stable (0.801, 0.804, 0.995), and then diverged sharply at R3–R4: the slope collapsed to 0.574 (R3) and 0.549 (R4), while the rank correlation stayed comparatively high (ρ 0.827 at R3, 0.736 at R4) (n = 144 at R0–R1, 146 at R2–R4; Figure S3A,B). Symmetric regression, which treats the two templates alike, gives the same picture with less attenuation: the C1 slope is 0.705 (SMA) and 0.654 (Deming) at R3, and 0.692 (SMA) and 0.632 (Deming) at R4, against 0.988–1.045 for C2 and 0.957–1.006 for C3 in every round. The defining bias is this R3–R4 slope collapse, not any departure at the early rounds. By contrast, the intermediate cluster C2 tracked the identity line across all rounds (ρ 0.893–0.977; slope 0.973–1.027; n = 62 at R2–R4), and the depleted cluster C3 likewise stayed on the identity line (ρ 0.974–0.986; slope 0.947–1.000; n = 580 at R2–R4). This divergence is directional: ssDNA over-reports the most enriched clonotypes while dsDNA under-counts them.

The template bias was also reflected in HCDR3 length during panning rounds. The read-weighted length distribution was concordant between templates in early rounds but split at R3–R4: ssDNA became bimodal, with modes at 12 aa (36–38% of reads) and 17 aa (19–24%), whereas dsDNA remained unimodal at 17 aa (33–41%) (Figure 2C). The most ssDNA-inflated clonotype (HCDR3 sequence GASGYSSGNIDA) belonged to the C1 cluster, showed a 34-fold higher fraction of reads in ssDNA than in dsDNA at R3 (20.6% vs. 0.61%), and accounted for the 12-aa length mode in ssDNA.

Abundance-weighted overlap between the two repertoires mirrored the correlation results, remaining near unity until selection concentrated the repertoire. The Morisita–Horn index, which, unlike rank correlation, is sensitive to the actual proportions in each frequency profile rather than just their order, was 0.997 (R0), 0.998 (R1), and 0.994 (R2) before falling sharply to 0.501 (R3) and 0.379 (R4) (Figure S3C). This drop happens at the same round as the collapse in C1’s slope, showing that the two templates report the same distributions until the enriched rounds.

This same C1-localized, directional bias appeared independently in the cluster assignments: of the 1003 shared clonotypes, each labeled independently from its ssDNA and dsDNA copies, 215 (21.4%) were discordant, and dsDNA assigned more of them to C1 than ssDNA did (279 vs. 160), almost entirely through transitions into C1 (93 enriching in dsDNA but depleted in ssDNA, against 1 in reverse; Figure S3D). Their exclusion is not what produces the result: restricting C1 to cluster-concordant clonotypes, to the dsDNA labels alone (n = 279), to the ssDNA labels alone (n = 160) or to their union (n = 293) gives R3/R4 slopes of 0.57/0.55, 0.62/0.62, 0.59/0.56 and 0.63/0.62, respectively, all far below unity, and including them moves no cluster at any round by more than 0.075 (Figure S3E). Among the assayed clonotypes that dsDNA assigns to C1, those ssDNA also assigns to C1 were antigen-reactive in 25 of 32 cases (78%), whereas those ssDNA assigns elsewhere were reactive in 2 of 17 (12%; Fisher exact test, *p* = 1.1 × 10^−5^; Figure S3F), consistent with an enriching call made on an almost flat dsDNA trajectory being a weak one: their dsDNA trajectories span about half the dynamic range of the concordant ones (0.08 versus 0.17 log_10_ units; *p* = 2 × 10^−40^). Taken together, these results separate two effects that are easy to conflate: whether the templates disagree on which clonotypes are enriched (rank), and on how enriched they are (abundance). Crucially, rank order was preserved throughout (C1 ρ ≥ 0.73, C2 ρ ≥ 0.89, and C3 ≈ 0.98) (Figure 2B and Figure S3A). Comparing each metric’s own point of maximal divergence relative to the R2 baseline, the abundance-weighted overlap fell ~62% (Morisita–Horn 0.99 → 0.38; Figure S3C) at R4, while the whole-library rank correlation remained high throughout (ρ ≥ 0.90; Figure S2); thus, template choice distorts clonal abundance, not rank.

**Figure 2:**
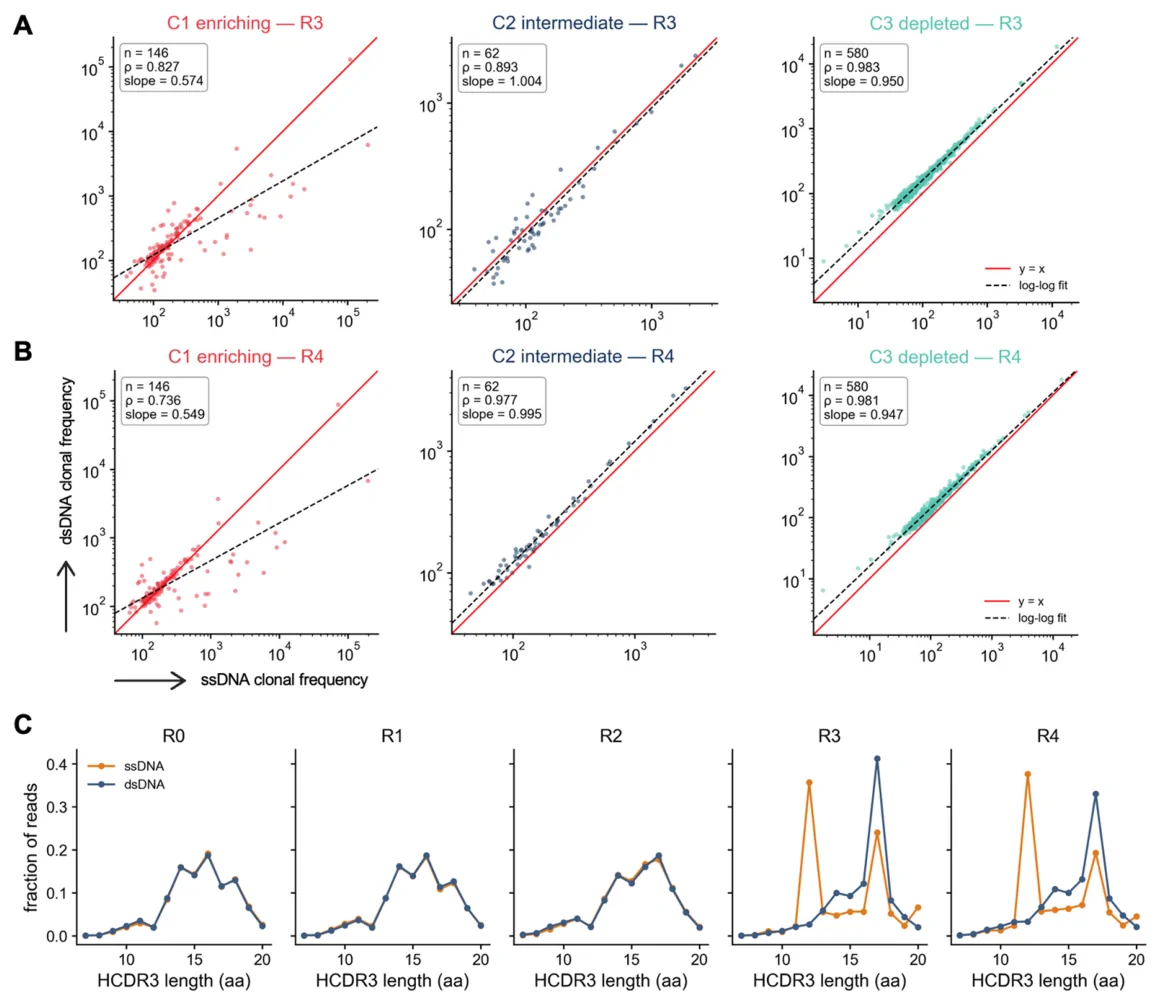
Frequency bias of clonotypes between the ssDNA and dsDNA repertoires. (**A**,**B**) Frequency bias of shared HCDR3 clonotypes between the ssDNA and dsDNA repertoires, colored by pooled cluster (C1, enriching; C2, intermediate; C3, depleted), at R3 (**A**) and R4 (**B**); red line, y = x; dashed line, ordinary-least-squares log–log fit; insets give n, Spearman ρ, and the log–log slope. (**C**) HCDR3 length distribution (fraction of reads) per round in the ssDNA and dsDNA repertoires.

### 3.4. Antigen-Reactive HCDR3 Clonotypes Are Enriched in the ssDNA-Biased Fraction

To examine how the template bias affects antigen reactivity, we used the phage-ELISA reactivity calls determined for the retrieved clones. Of the 568 unique HCDR3 sequences (90 antigen-reactive and 478 non-reactive determined by majority vote, as described in Methods), 146 were detected in both template analysis sets (35 ELISA-positive and 111 ELISA-negative) (Table S1). Among these assayed, the antigen-reactive HCDR3 clonotypes sat preferentially on the ssDNA-favored side of the identity line. Overlaying these on the per-round ssDNA-versus-dsDNA clonal-frequency scatter against the 1003 shared clonotypes showed that the ELISA-positive clonotypes were displaced toward the ssDNA repertoire at the enriched rounds (Figure 3A). Through R2 the ELISA-positive and -negative clonotypes were interspersed along the identity line (Figure 3A, R0–R2), and the ssDNA displacement of the antigen-reactive clonotypes emerged only at R3–R4. Quantifying this at R3, the distribution of log_10_(ssDNA/dsDNA) had a median of +0.44 for the 35 ELISA-positive clonotypes versus −0.17 for the 111 ELISA-negative clonotypes (two-sided Mann–Whitney *U* test, *p* = 6.3 × 10^−8^) (Figure 3B), and +0.41 vs. −0.12 at R4 (*p* = 1.1 × 10^−6^; Figure S4). A median log-ratio of +0.44 corresponds to roughly 2.8-fold higher abundance in the ssDNA repertoire for the antigen-reactive group, reflecting greater ssDNA quantification of these clonotypes rather than their detectability. Across all rounds the two groups were indistinguishable through R2 (difference in medians +0.02 at R0, +0.03 at R1 and −0.03 at R2) and separated only at R3 and R4 (Figure S4). Because these clonotypes come from a single bio-panning campaign and are not independent biological replicates, these *p* values describe this library rather than supporting population-level inference. Notably, the most ssDNA-inflated clonotype with the HCDR3 sequence of GASGYSSGNIDA is ELISA-positive, tying the abundance bias directly to a validated antigen-binder.

This is an enrichment of antigen-reactive clones within the ssDNA-biased fraction, not evidence that the template bias predicts reactivity. Because only HCDR3-matched clonotypes were available and there is no dsDNA-biased control group, the result establishes that antigen-reactive clonotypes are overrepresented among the ssDNA-favored clones, but it does not show that the ssDNA/dsDNA ratio can be used to predict reactivity.

The practical consequence is that template choice biases the quantification of enriched (including antigen-reactive) clonotypes: ssDNA captures their abundance more completely, whereas dsDNA underrepresents the most enriched clones.

Notably, physicochemical features (Atchley factor) and ESM-2-based sequence embeddings did not separate antigen-reactive from non-reactive clonotypes (linear-probe area under the receiver-operating-characteristic curve [AUC] of 0.53 for Atchley factors and 0.55 for ESM-2 embeddings; Figure S5); these representations, used together with a linear classifier, therefore did not separate the two groups in this dataset, which bounds what they can detect here but does not establish that the HCDR3 sequence carries no information about reactivity.

**Figure 3:**
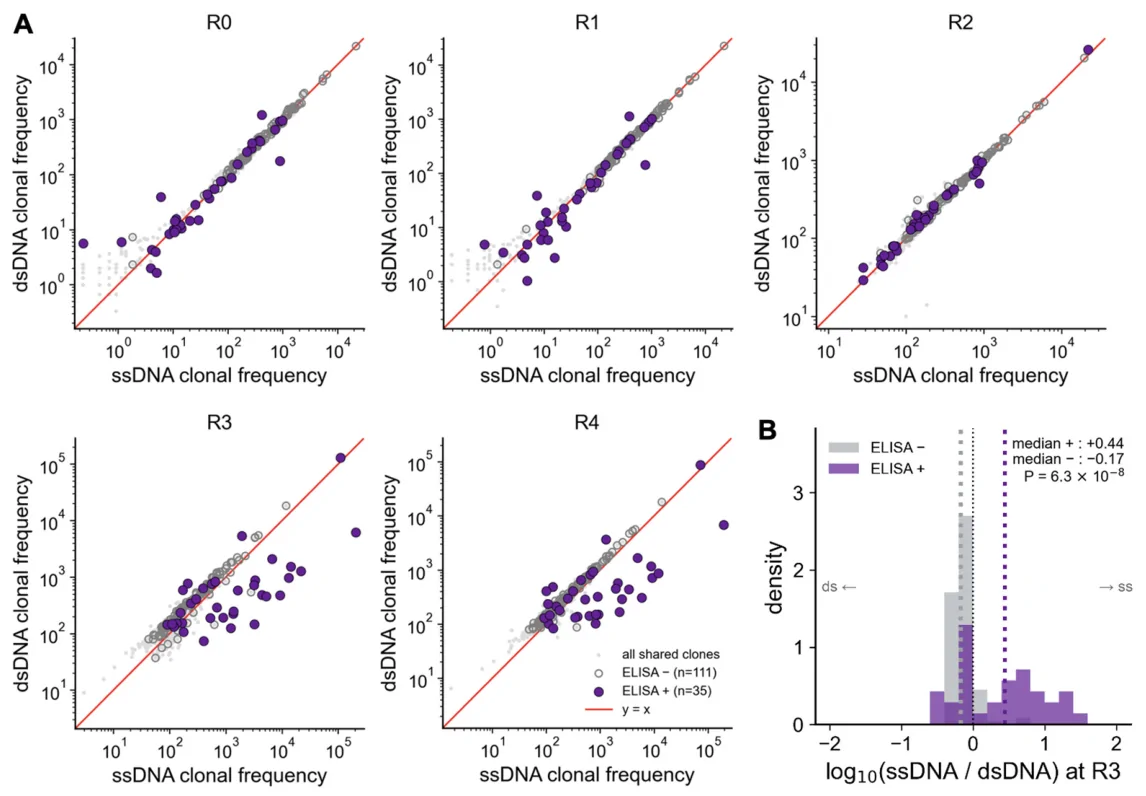
Antigen-reactive HCDR3 clonotypes are enriched in the ssDNA-biased fraction. (**A**) Frequency of all shared HCDR3 clonotypes (grey dots) in the ssDNA and dsDNA repertoires, per round (R0–R4). Clonotypes assayed by phage ELISA are overlaid as antigen-reactive (purple circles, ELISA +, n = 35) or non-reactive (white circles, ELISA −, n = 111). Red line, y = x. (**B**) Distribution of log_10_(ssDNA/dsDNA) at R3 for antigen-reactive (ELISA +, n = 35) and non-reactive (ELISA −, n = 111) HCDR3 clonotypes (vertical lines, medians; *p*, two-sided Mann–Whitney U test).

## 4. Discussion

In this work, we found that the ssDNA and dsDNA repertoires agreed closely in rank correlation and clonal diversity through R0–R2 and recovered the same overall three-trajectory cluster structure, but diverged specifically within the enriching cluster (C1) at the enriched rounds (R3–R4). The ssDNA repertoire overrepresented the most abundant C1 clonotypes relative to their dsDNA counterparts, while the intermediate (C2) and depleted (C3) clusters remained concordant between templates throughout. Notably, these ssDNA-biased clonotypes were themselves enriched for antigen reactivity. This pattern was reflected quantitatively: the rank correlation between ssDNA and dsDNA (Spearman ρ) remained relatively high across all rounds (falling by at most ~8%), whereas the abundance-weighted overlap (Morisita–Horn index) and the log–log slope both collapsed sharply at R3–R4 (Morisita–Horn falling by ~62%). Thus, the two templates consistently agreed on which clonotypes survived bio-panning (rank) but disagreed sharply on how enriched each clonotype actually was (abundance).

A central observation of this study is that antigen-reactive clonotypes were preferentially displaced toward the ssDNA (virion) repertoire at the enriched rounds. One explanation consistent with this pattern is that antigen-reactive clonotypes are packaged into virions more efficiently than non-reactive ones as bio-panning progresses, although our data establish only an association and do not demonstrate this mechanism. There are mechanisms reported in the literature that can account for this phenomenon.

First, phage assembly imposes a folding and secretion filter on scFv-pIII fusion proteins. Only correctly folded fusion proteins are efficiently translocated to the periplasm and incorporated into the virion as pIII fusions, whereas misfolded or unstable variants are degraded or fail to assemble into infective particles [35]. Even proteins that fold well intracellularly are displayed poorly when their translocation into the periplasm is inefficient, so secretion competence governs how much of a clone is packaged into phage and how strongly it is enriched per round [36]. If this filtering effect intensifies over successive rounds of bio-panning, the antigen-reactive clones surviving the late rounds would increasingly be the well-folded, secretion-competent ones, and these would come to dominate the virion (ssDNA) repertoire.

Second, the level at which an antibody fragment is displayed—its valency—scales with how well the fragment is expressed, which in turn favors efficient phage production. Display propensity correlates strongly with overall scFv expression [37], so well-expressed binders are presented at higher valency and incorporate more fusion protein per particle; this higher-valency display also confers a direct selection advantage through more avid antigen binding during bio-panning. Consistent with this, selection formats that favor multivalent display co-enrich clones with higher expression, thermostability, and affinity [38]. On this account, the same physicochemical properties that make a clonotype a strong, selectable binder would also make it a more prolific producer of displaying phage, biasing such clones toward the packaged ssDNA fraction.

Collectively, these mechanisms offer one plausible explanation for why antigen-reactive clones might be over-produced as virions at the late rounds—that they are the clones best able to fold, be secreted, be displayed at high density, and be packaged—but none of these properties was measured here, and the account is therefore drawn from the cited literature rather than tested by our data. Whether these effects act at the packaging interface rather than at the level of intracellular phagemid replication cannot be resolved from our data; separating them would require per-clone measurements of phage production and packaging efficiency, which we did not perform. What the data do show is that the two templates diverge only once binders come to dominate the selection, that the divergence is restricted to the enriching clonotypes, and that the antigen-reactive clonotypes among them sit preferentially on the ssDNA side of that divergence.

In peptide display, packaging efficiency is itself a sequence-dependent property. Paired-end and deep-sequencing analyses of phage-displayed libraries reveal a compositional packaging bias in which particular sequences are funneled preferentially into virions [11,12]. But in our study, HCDR3 physicochemical features (Atchley factors) and ESM-2-based sequence embeddings did not predict antigen reactivity: a linear probe inferred through these features achieved an AUC of 0.53 (Atchley factors) and 0.55 (ESM-2 embeddings), close to chance level (Figure S5). This bounds what these particular representations and a linear classifier can detect in 562 sequences; it does not establish that the HCDR3 sequence carries no information about antigen reactivity.

Deep sequencing has become a standard part of antibody phage-display workflows [39]: it is used to profile libraries before selection [40], to mine selection outputs at depth with long reads and unique molecular identifiers [41], and to support the construction of very large synthetic libraries [42]. The template from which those reads are generated is therefore a choice that affects a widely used pipeline. Our findings also offer practical guidance for antibody discovery. Selecting candidates for further development solely on the basis of their abundance in the dsDNA repertoire can deprioritize clonotypes that are dominant in the ssDNA repertoire. For example, the most ssDNA-inflated clonotype with the HCDR3 sequence of GASGYSSGNIDA showed comparatively modest abundance in the dsDNA repertoire, illustrating a practical false-negative risk in which a genuine binder is overlooked when candidates are selected from dsDNA-based data alone.

Moreover, when clonotypes were clustered on the basis of dsDNA sequence, more clonotypes were assigned to C1 by dsDNA than by ssDNA (279 vs. 160; 21.4% discordant) (Figure S3D). This suggests that the dsDNA-defined C1 may contain two distinct groups: true-positive clonotypes enriched through genuine antigen specificity, and clonotypes that appear enriched primarily because of a bacterial growth advantage, independent of antigen-binding affinity. In terms of antibody discovery, this raises a complementary concern—a false-enriching problem—in which some clones that are not antigen-reactive may be mistakenly prioritized when candidates are screened solely from dsDNA-based HTS data. In addition, the converse error is equally possible: because enriched clonotypes reach a lower relative abundance in dsDNA than in ssDNA, genuine binders may be misclassified into the intermediate or depleted clusters and overlooked. Consistent with this, among the assayed clonotypes that dsDNA assigns to C1, antigen reactivity was confirmed in 78% (25/32) of those that ssDNA also assigns to C1 but in only 12% (2/17) of those it does not (Figure S3F); this comparison derives from the same ssDNA/dsDNA abundance difference as Figure 3 and is therefore not independent of it.

This study has two limitations. First, generalizing our findings will require testing across various antigens, different scaffolds (e.g., Fab or VHH), and other phagemid display systems (e.g., pVIII). Second, only 146 of the 568 assayed HCDR3 sequences were matched between the two template analysis sets, and the absence of a dsDNA-biased control group means we can establish only an association, not a causal relationship, between ELISA-positive signals (antigen reactivity) and ssDNA bias.

In summary, we showed that dsDNA-based analysis can underestimate the phage output captured in the ssDNA (virion) repertoire of enriched, antigen-reactive clonotypes at the late rounds of bio-panning. To our knowledge, this is the first large-scale, quantitative demonstration of this genotype (dsDNA)–phenotype (ssDNA) divergence across an entire bio-panning campaign.

## 5. Conclusions

Sequencing the phagemid recovered from infected bacteria and the phagemid packaged into virions gives the same account of a bio-panning campaign while the library remains diverse, and different accounts once selection concentrates it. Through the second round, the two templates agreed on both the ranking and the proportions of HCDR3 clonotypes. At the third and fourth rounds, the ranking was still preserved, but the abundance-weighted overlap between the two repertoires fell by more than half, and the divergence was confined to the clonotypes that the selection was enriching, with antigen-reactive clonotypes sitting preferentially on the ssDNA-favored side of that divergence. For work that reads candidate priority from late-round abundance, virion ssDNA—or the two templates analyzed together—is therefore the safer basis; for work that reads only the rank order of clonotypes, either template will serve. These conclusions describe a single campaign against a single antigen and will need testing across other antigens, antibody scaffolds and display formats.

## Data Availability

The raw sequencing data generated in this study have been deposited in the NCBI Sequence Read Archive (SRA) under BioProject accession number PRJNA1507317. All analysis code, together with the derived tables needed to reproduce every reported value, is available at https://github.com/dk93js/phage-sequencing (accessed on 14 September 2026).

## Supplementary Materials

The following supporting information can be downloaded at: https://www.mdpi.com/article/10.3390/biom16101379/s1, Figure S1. High-throughput sequencing (HTS) statistics, HCDR3 clonotype enrichment, and clustering analysis. Figure S2. Frequency correlation of HCDR3 clonotypes between the ssDNA and dsDNA repertoires per round (R0–R4). Figure S3. Clonal overlap and rank correlation between the ssDNA and dsDNA repertoires by enrichment cluster, and the clonotypes the two templates assign differently. Figure S4. Antigen-reactive versus non-reactive log10(ssDNA/dsDNA) at every round. Figure S5. Sequence features of antigen-binding and non-binding HCDR3 clonotypes. Table S1. Phage-ELISA calls for the HCDR3 clonotypes present in both template analysis sets.

## Abbreviations

The following abbreviations are used in this manuscript:

ssDNA	Single-stranded DNA
dsDNA	Double-stranded DNA
HCDR3	Heavy-chain complementarity-determining region 3
scFv	Single-chain variable fragment
E. coli	Escherichia coli
HTS	High-throughput sequencing
VH	Heavy-chain variable region
JH	Heavy-chain joining region
FR3	Framework region 3
FR4	Framework region 4
PPM	Parts per million
TR	TrueRepertoire
SMA	Standardized major axis
SB	Super Broth
BSA	Bovine serum albumin
PBS	Phosphate-buffered saline
PBST	Phosphate-buffered saline with Tween-20
HRP	Horseradish peroxidase
TMB	3,3′,5,5′-Tetramethylbenzidine
ELISA	Enzyme-linked immunosorbent assay
SD	Standard deviation
GRAVY	Grand average of hydropathicity
UMAP	Uniform Manifold Approximation and Projection
AUC	Area under the (receiver-operating-characteristic) curve
NRF	National Research Foundation
MSIT	Ministry of Science and ICT
KHIDI	Korea Health Industry Development Institute
SRA	Sequence Read Archive
